# The Influence of Computed Tomography on the Preoperative Planning of Revision Hip Arthroplasty – Femoral Component

**DOI:** 10.1055/s-0045-1810034

**Published:** 2025-09-08

**Authors:** Rafaela Reis Torrealba, Maria Isabella Cruz de Castro, Phercyles Veiga-Santos, Marcelo Felipe Almeida, Conrado Torres Laett, Lourenço Peixoto

**Affiliations:** 1 Instituto Nacional de Traumatologia e Ortopedia Jamil Haddad, Rio de Janeiro, RJ, Brazil.

**Keywords:** arthroplasty, replacement, hip, revision surgery, surgery, orthopedic, tomography, x-ray computed, artroplastia de quadril, cirurgia de revisão, cirurgia ortopédica, tomografia computadorizada

## Abstract

**Objective:**

The present study aimed to compare the accuracy of the Paprosky Classification of Femoral Bone Loss using plain radiographs and two-dimensional computed tomography (2D CT) images with the femoral defect observed intraoperatively by the surgeon.

**Methods:**

There were 14 hip surgeons from the same hospital who classified 80 patients with an indication for revision hip arthroplasty according to Paprosky based on plain radiographs in anteroposterior views of the pelvis and 2D CT images, reconstructed in the axial, coronal, and sagittal planes. We compared this data with the intraoperative findings of femoral bone loss by the same surgeons.

**Results:**

The agreement between the radiographic and CT assessment was excellent for femoral bone defects (94% agreement; κ = 0.95; 0.90–0.99). Individually, the radiograph-based classification agreed with the intraoperative classification in 85% of cases (κ = 0.8; 0.70–0.90). The CT-based one had 86% of agreement (κ = 0.84; 0.75–0.93). There was no statistical difference between the methods.

**Conclusion:**

The use of 2D CT did not show any benefits in recognizing femoral bone loss by the Paprosky classification compared with radiography. Therefore, the significance of 2D images in planning femoral component revision surgery should be questioned, as it is associated with higher financial costs and greater patient exposure to high radiation levels.

## Resumo

**Objetivo:**

Este estudo teve como objetivo comparar a acurácia da Classificação de Paprosky de Perda Óssea Femoral utilizando radiografias simples e imagens de tomografia computadorizada bidimensional (TC-2D), com o defeito femoral observado no intraoperatório pelo cirurgião.

**Métodos:**

Um total de 80 pacientes com indicação para a revisão de artroplastia do quadril foram classificados de acordo com Paprosky, por 14 cirurgiões do quadril do mesmo hospital, com base em radiografias simples em anteroposterior da pelve e posteriormente baseadas emTC-2D, reconstruídas nos planos axial, coronal e sagital. Esses dados foram comparados com os achados de perda óssea femoral no intraoperatório pelos mesmos cirurgiões.

**Resultados:**

A concordância entre a avaliação feita por radiografia e TC é excelente para o defeito ósseo femoral (94% de concordância; κ = 0,95; 0,90–0,99). Individualmente, a classificação feita de acordo com a radiografia concorda com a classificação intraoperatória em 85% dos casos (κ = 0,8; 0,70–0,90). Enquanto a feita com base na TC tem 86% de concordância (κ = 0,84; 0,75–0,93). Não houve diferença estatística entre os métodos.

**Conclusão:**

O uso da TC-2D não mostrou benefícios no reconhecimento da perda óssea femoral pela classificação de Paprosky em comparação com a radiografia. Portanto, deve-se questionar a importância das imagens 2D no planejamento da cirurgia de revisão do componente femoral, visto que se associa com maior custo financeiro e à maior exposição do paciente a níveis elevados de radiação.

## Introduction

Total hip arthroplasty (THA) is an extremely effective procedure for treating hip osteoarthritis. The number of revision surgeries has increased due to the increased amount of THAs performed, the decreased age of patients undergoing surgery, and the increased life expectancy. Estimates report that the need for surgery will exceed 130% by 2030. 1 2 3

Indications for revision THA (rTHA) include osteolysis, instability, aseptic loosening, fractures, infections, and wear. 3 4 All causes for revision involve bone loss to varying degrees. Preoperative planning must be meticulous since this is a highly complex surgery.

The Paprosky classification, based on pre- or intraoperative imaging evaluation, allows surgeons to plan the best implant and technique. 5 6 This classification for femoral bone loss was developed in 1994 and includes four progressive defect stages. Type I corresponds to a minimal metaphyseal defect and II to an extensive metaphyseal defect with minimal diaphyseal loss. Type III is divided into A and B, with IIIA corresponding to an extensive metaphyseal and diaphyseal defect, with an intact diaphysis up to ≥ 4 cm from the isthmus; and type IIIB representing an extensive metaphyseal and diaphyseal defect, with the diaphysis intact up to < 4 cm from the isthmus. Type IV defects are the most severe and consist of an extensive metaphyseal and diaphyseal defect with an insufficient isthmus. 6 The extensive literature documents that plain radiographs and computed tomography (CT) scans ensure accuracy and reproducibility and minimize intraoperative complications, improving the success rate of revision surgery.

The present study aimed to compare the accuracy of the Paprosky classification using plain radiographs and two-dimensional (2D) CT images with the femoral defect observed intraoperatively by the surgeon. Our aim is to ensure a safe and technically reproducible preoperative planning for femoral component revision surgery.

## Materials and Methods

This observational and prospective study followed up 80 patients with an indication for revision hip arthroplasty from January 2021 to December 2022. The inclusion criteria were patients aged over 30 years and radiographs showing aseptic failure of the femoral component. We excluded those with periprosthetic fractures or active infections.

There were 14 senior (> 10 years) and junior (< 10 years) surgeons, members of the Specialized Hip Center of our hospital, who assessed all patients according to the Paprosky classification ( Table 1 ) based on plain anteroposterior radiographs of the pelvis and 2D CT images reconstructed in the axial, coronal, and sagittal planes. We extracted these images from the hospital's medical archive using the MDICON software.

**Table 1:** Paprosky classification of femoral bone loss

Type	Description
I	Minimal metaphyseal defect
II	Extensive metaphyseal defect, with minimal diaphyseal loss
IIIA	Extensive metaphyseal and diaphyseal defect, intact diaphysis up to ≥ 4 cm from the isthmus
IIIB	Extensive metaphyseal and diaphyseal defect, intact diaphysis up to < 4 cm from the isthmus
IV	Extensive metaphyseal and diaphyseal defect, insufficient isthmus

Surgical planning included the evaluation of the most appropriate femoral implant, the need for bone grafting, and the presence or absence of pelvic discontinuity. A new assessment occurred 30 days after the first, following the same classification and surgical planning criteria. We randomly changed the order of the images in the Excel (Microsoft Corp.) 2019 program. There was no identification of the medical records or patients' names. The intraoperative classification of femoral bone loss was the gold standard.

All surgeons and patients signed the informed consent form (ICF). The Research Ethics Committee approved the study under opinion No. 003760/2021 and CAAE No. 42181421.5.0000.5273.

We expressed the classification agreement in percentage values and the kappa (κ) coefficient, as follows: poor (< 0.20), weak (> 0.20 and < 0.40), moderate (> 0.40 and < 0.60), good (> 0.60 and < 0.80), and excellent (> 0.80), presenting a 95%CI per Landis and Koch. 7 The κ value comparison was determined by the CI, and statistical differences occurred when there was no overlap.

## Results

The agreement between the radiograph- and CT-based assessment was excellent for the femoral bone defect (94% agreement; κ = 0.95; 0.90–0.99) as shown in Table 2 .

**Table 2:** Confusion matrix for femoral bone defect classification

		Computed tomography				
		1	2	3A	3B	4
**Radiography**	**1**	28	0	0	0	0
	**2**	0	29	2	0	0
	**3A**	0	0	20	1	0
	**3B**	0	0	0	8	0
	**4**	0	0	0	0	2

**Fig. 1:**
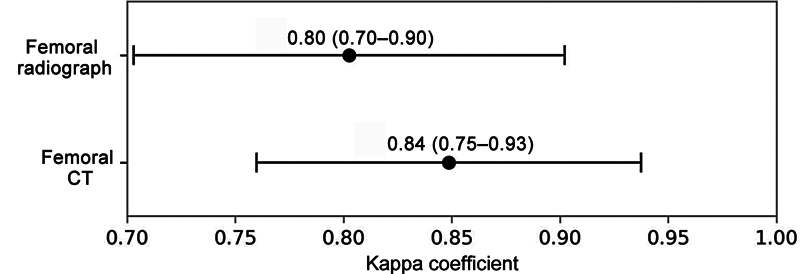
Kappa coefficient values (95%CI) for agreement assessment in the Paproski classification of hip bone defects. **Abbreviation:** CT, computed tomography.

The radiograph-based classification agreed with the intraoperative classification in 85% of cases. The CT-based one had 86% rate of agreement. There was no statistical difference between the methods ( Fig. 1 ).

The agreement of surgical planning based on radiographic and TC images was complete. We observed a 90% agreement between the surgical planning and the procedure performed in the femoral component (κ = 0.74; 0.59–0.89).

## Discussion

The Paprosky classification is a tool assisting in the preoperative planning of THA revision surgery. Additionally, it can quantify bone loss of the femoral component based on imaging and help select the best implant and technique. Thus, the classification of predetermined bone defects must be faithful to intraoperative findings.

A useful classification must present good interobserver and intraobserver reliability. Brown et al. 8 asked four hip surgeons to evaluate femoral bone loss using the Paprosky classification of 205 radiographs of patients before revision. These images were randomly displayed one month later for all surgeons for a new classification. The authors found substantial interobserver reliability (0.61) and consistent intraobserver agreement (0.81, 0.78, 0.76, and 0.75).

Our study aimed to analyze the predictive value of the Paprosky classification based on plain radiography and 2D CT for preoperative planning. In practice, a high predictive value means the ability to plan and select the most probable implant, and the type or amount of bone graft, when required. 9 We confirmed that the Paprosky classification for femoral bone loss has substantial agreement between the preoperative planning and the actual procedure. However, we found no significant difference between radiography and 2D CT models.

The literature has few articles evaluating the accuracy of preoperative planning using radiography and 2D CT in patients with femoral bone loss. To date, most authors have assessed these data in primary THA. Many studies have demonstrated that three-dimensional (3D) CT models present excellent reliability regarding the size and alignment of the planned components. 10 11 12 Sariali et al. 13 and Reinbacher et al. 14 compared 2D and 3D models to define the size of the femoral cup and stem for implantation and concluded that 3D models are superior in efficiency, accuracy, and reproducibility.

However, the literature regarding revision surgery planning, particularly femoral component revision, is scarce. Most studies envisaged the acetabular component revision. Winter et al. 15 evaluated the accuracy of preoperative planning through 3D impressions compared with 2D in 27 patients requiring acetabular reinforcement cages (Burch Schneider). These authors compared the planned implant sizes with the final sizes. The customized 3D templates predicted the exact implant size in 96.3% of patients, compared with only 55.6% of 2D. Although this finding strongly suggests that 3D reconstructions are more reliable, it is worth noting that the included patients had massive bone defects, that is, Paprosky type IIA-IIIB, and the authors excluded subjects with minor bone loss. Furthermore, the study did not clarify the surgeon's experience level.

Plate et al. 16 compared the Paprosky classification for acetabular bone loss in eight THA revisions based on plain radiographs, 2D CTs, and 3D reconstructions. The images were reviewed by 35 first-year residents, 2 fellows, and 2 orthopedic surgeons with the responses being compared to those from 2 expert hip surgeons. Radiography increased the number of correct classifications (0.37), while 2D CT and 3D reconstructions did not improve accuracy (0.33, 0.20; *p* < 0.001), contradicting most existing studies. The authors emphasized that the surgeons' experience level did not influence the correct Paprosky classification.

Some authors have evaluated the reliability of other bone loss classification systems based on radiographs. Käfer et al. 9 asked two hip surgeons to assess radiographs from 33 patients regarding the bone defect and decide the best implant and technique for each case. Another examiner verified whether the surgeon would perform the planned procedure. They used the Saleh classification. The analysis between the preoperative radiological estimates and the intraoperative conclusions regarding the implant and the bone graft revealed correlation coefficients of 0.63 ( *p* < 0.01) for the femoral classification. It can be argued that the difference between the Paprosky and Saleh classifications is that the former considers the visible radiographic defect and the one likely to be found after implant removal. Additionally, the number of patients participating in their study was smaller than ours.

The present study had some limitations. We classified hip surgeons as juniors when they had less than 10 years of experience, and as seniors those with more than 10 years. In our opinion, the experience time as a hip surgeon may interfere with the accuracy of the pre- and intraoperative Paprosky classification. Although we have proven that preoperative planning with the aid of 2D CT does not present any benefits over radiography, 3D models are the gold standard. Despite this, we did not use this resource as a basis for comparison.

## Conclusion

There were no benefits for 2D CT use in recognizing femoral bone loss according to the Paprosky classification. Furthermore, in these cases, this imaging technique has higher costs than radiography and greater patient exposure to high radiation levels. Therefore, one must question the role of CT in planning femoral component for rTHA.
